# Supplementary material for: Relationships between different components of intolerance of uncertainty and symptoms of obsessive–compulsive disorder: a network analysis
Source: PeerJ. 2025 Jul 31;13:e19791. doi: 10.7717/peerj.19791 (PMC12318506; doi:10.7717/peerj.19791)
Supplement: Supplemental Information 2 [file peerj-13-19791-s002.docx]

The results of IU-OCD network Supplementary Materials

1. Table S1. The Correlation matrix between components of IU and symptoms of OCD

2. Figure S1. Accuracy of edge weights

3. Figure S2. Bootstrapped difference test for edge weights.

4. Figure S3. Bootstrapped difference test for node expected influences

5. Figure S4. Bootstrapped difference test for node bridge expected influences

6. Figure S5. The network structures generated from the two genders.

7. Figure S6. The network structure invariance and network global strength between males and females

Table S1. The Correlation matrix between components of IU and symptoms of OCD

|  | | IU1 | IU2 | IU3 | IU4 | IU5 | IU6 | IU7 | IU8 | IU9 | IU10 | IU11 | IU12 |
| --- | --- | --- | --- | --- | --- | --- | --- | --- | --- | --- | --- | --- | --- |
|  | OCD1 | .524^**^ | .525^**^ | .516^**^ | .301^**^ | .501^**^ | .544^**^ | .501^**^ | .372^**^ | .499^**^ | .496^**^ | .177^**^ | .363^**^ |
|  | OCD2 | .463^**^ | .442^**^ | .470^**^ | .402^**^ | .425^**^ | .452^**^ | .472^**^ | .402^**^ | .424^**^ | .416^**^ | .307^**^ | .423^**^ |
|  | OCD3 | .572^**^ | .543^**^ | .563^**^ | .434^**^ | .528^**^ | .547^**^ | .544^**^ | .444^**^ | .532^**^ | .499^**^ | .322^**^ | .480^**^ |
|  | OCD4 | .403^**^ | .452^**^ | .447^**^ | .230^**^ | .441^**^ | .446^**^ | .417^**^ | .255^**^ | .456^**^ | .476^**^ | .134^**^ | .368^**^ |
|  | OCD5 | .463^**^ | .473^**^ | .503^**^ | .259^**^ | .449^**^ | .466^**^ | .457^**^ | .266^**^ | .465^**^ | .476^**^ | .166^**^ | .394^**^ |
|  | OCD6 | .506^**^ | .531^**^ | .541^**^ | .237^**^ | .526^**^ | .558^**^ | .510^**^ | .319^**^ | .574^**^ | .574^**^ | .123^**^ | .433^**^ |
|  | OCD7 | .393^**^ | .429^**^ | .431^**^ | .206^**^ | .401^**^ | .406^**^ | .370^**^ | .274^**^ | .435^**^ | .444^**^ | .106^**^ | .348^**^ |
|  | OCD8 | .304^**^ | .315^**^ | .339^**^ | .234^**^ | .304^**^ | .324^**^ | .332^**^ | .264^**^ | .336^**^ | .323^**^ | .192^**^ | .348^**^ |
|  | OCD9 | .563^**^ | .558^**^ | .550^**^ | .369^**^ | .514^**^ | .563^**^ | .529^**^ | .410^**^ | .566^**^ | .517^**^ | .275^**^ | .496^**^ |
|  | OCD10 | .325^**^ | .360^**^ | .377^**^ | .157^**^ | .362^**^ | .377^**^ | .319^**^ | .195^**^ | .391^**^ | .427^**^ | .090^**^ | .350^**^ |
|  | OCD11 | .343^**^ | .362^**^ | .374^**^ | .197^**^ | .370^**^ | .374^**^ | .361^**^ | .239^**^ | .388^**^ | .419^**^ | .140^**^ | .376^**^ |
|  | OCD12 | .506^**^ | .537^**^ | .530^**^ | .344^**^ | .494^**^ | .535^**^ | .500^**^ | .358^**^ | .525^**^ | .521^**^ | .228^**^ | .461^**^ |
|  | OCD13 | .366^**^ | .368^**^ | .363^**^ | .376^**^ | .325^**^ | .343^**^ | .396^**^ | .369^**^ | .338^**^ | .315^**^ | .333^**^ | .349^**^ |
|  | OCD14 | .316^**^ | .312^**^ | .343^**^ | .309^**^ | .328^**^ | .327^**^ | .358^**^ | .334^**^ | .329^**^ | .329^**^ | .272^**^ | .320^**^ |
|  | OCD15 | .412^**^ | .418^**^ | .426^**^ | .337^**^ | .399^**^ | .417^**^ | .430^**^ | .337^**^ | .431^**^ | .432^**^ | .280^**^ | .427^**^ |
|  | OCD16 | .368^**^ | .384^**^ | .383^**^ | .190^**^ | .394^**^ | .415^**^ | .379^**^ | .273^**^ | .391^**^ | .421^**^ | .134^**^ | .358^**^ |
|  | OCD17 | .330^**^ | .352^**^ | .363^**^ | .164^**^ | .381^**^ | .397^**^ | .356^**^ | .198^**^ | .432^**^ | .419^**^ | .108^**^ | .314^**^ |
|  | OCD18 | .375^**^ | .431^**^ | .427^**^ | .185^**^ | .373^**^ | .426^**^ | .376^**^ | .260^**^ | .433^**^ | .444^**^ | .097^**^ | .338^**^ |
| **. P<0.01 | | | | | | | | | | | | | |


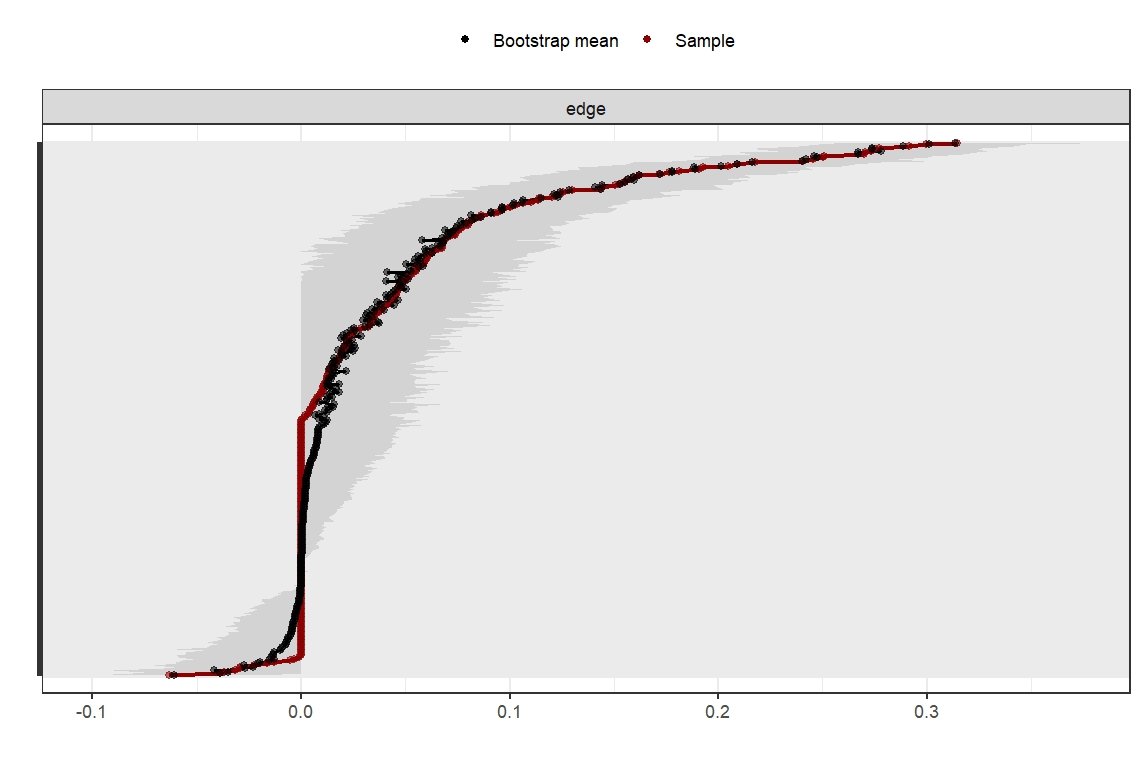


Figure S1. Accuracy of edge weights

Note: The red line depicts the sample edge weights and the gray bar depicts the bootstrapped confidence interval.


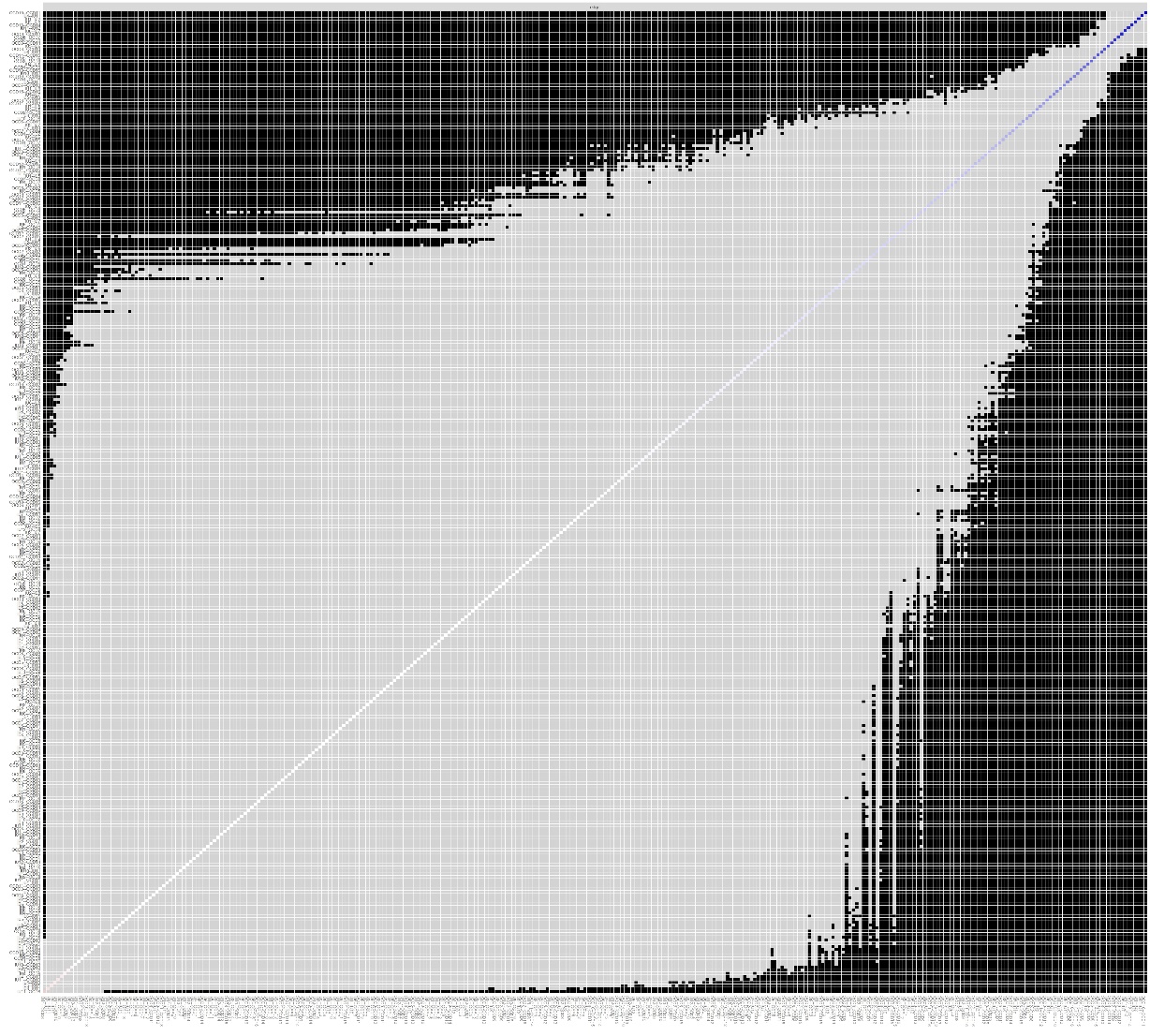


Figure S2. Bootstrapped difference test for edge weights.

Gray boxes indicate edge weights that do not differ significantly from one another, while black boxes indicate edge weights that do differ significantly.


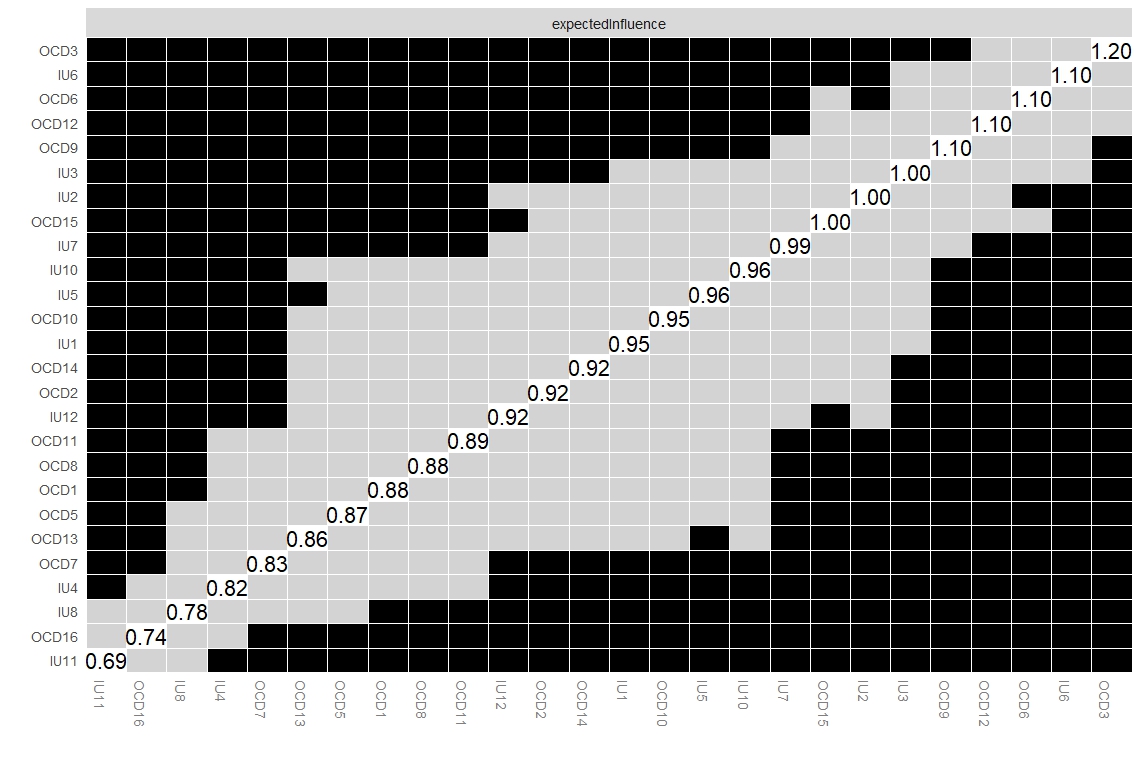


Figure S3. Bootstrapped difference test for node expected influences

Note: Gray boxes indicate node expected influences that do not differ significantly from one another, while black boxes indicate node expected influences that do differ significantly.


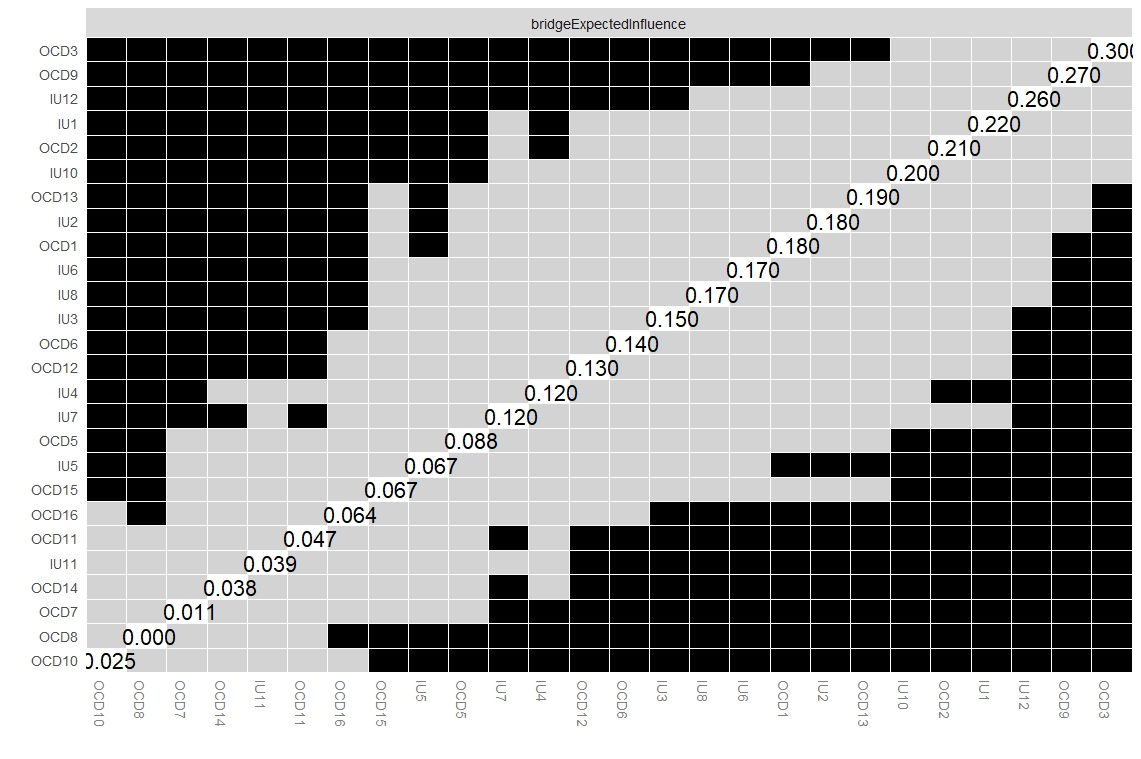


Figure S4. Bootstrapped difference test for node bridge expected influences

Note: Gray boxes indicate node bridge expected influences that do not differ significantly from one another, while black boxes indicate node bridge expected influences that do differ significantly.


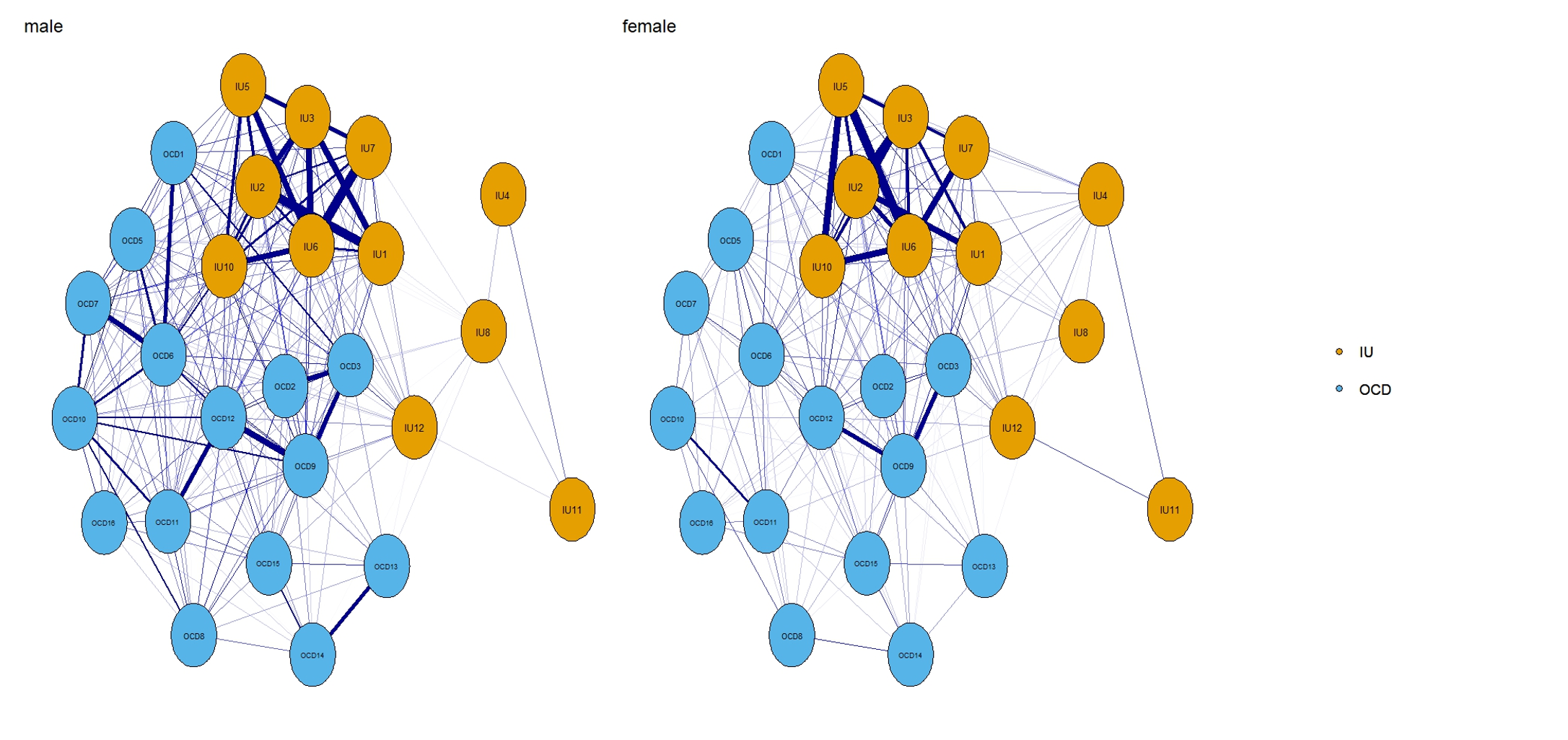


Figure S5. The network structures generated from the two genders.


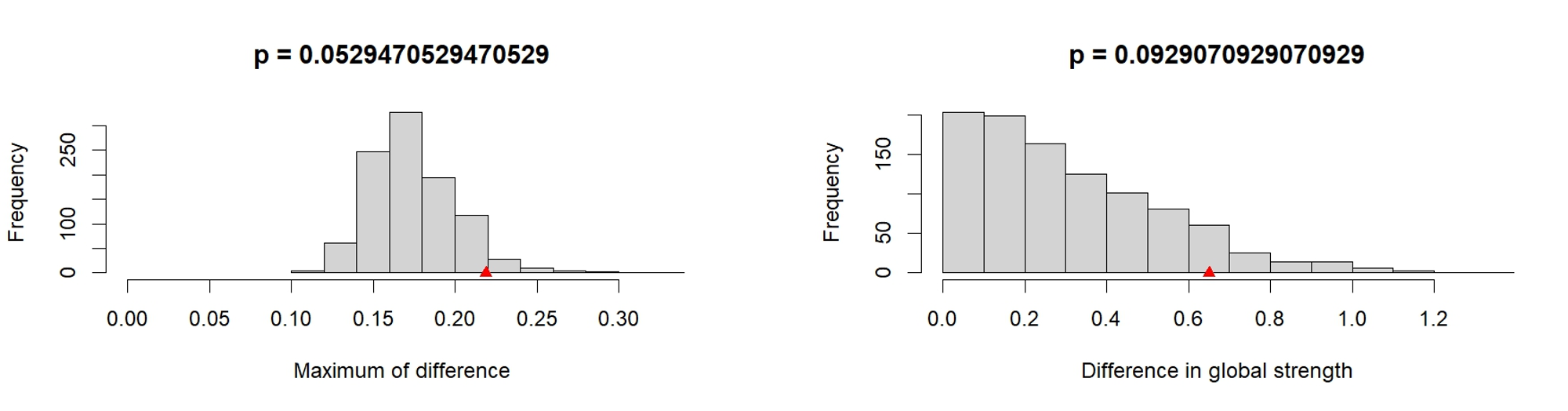


Figure S6. The network structure invariance and network global strength between males and females.
